# Photodynamic therapy combined to cisplatin potentiates cell death responses of cervical cancer cells

**DOI:** 10.1186/s12885-017-3075-1

**Published:** 2017-02-10

**Authors:** Laura Marise de Freitas, Rodolfo Bortolozo Serafim, Juliana Ferreira de Sousa, Thaís Fernanda Moreira, Cláudia Tavares dos Santos, Amanda Martins Baviera, Valeria Valente, Christiane Pienna Soares, Carla Raquel Fontana

**Affiliations:** 1 Universidade Estadual Paulista (Unesp), Faculdade de Ciências Farmacêuticas, Araraquara– Rod Araraquara-Jau km 01 s/n, Araraquara, Sao Paulo 14800-903 Brazil; 2 Faculdade de Medicina de Ribeirao Preto, USP Univ de Sao Paulo, Avenida dos Bandeirantes 3900, Ribeirao Preto, Sao Paulo 14049-900 Brazil

**Keywords:** Photodynamic therapy, Methylene blue, Photogem, Cisplatin, Combined therapy, Caspase-independent cell death

## Abstract

**Background:**

Photodynamic therapy (PDT) has proven to be a promising alternative to current cancer treatments, especially if combined with conventional approaches. The technique is based on the administration of a non-toxic photosensitizing agent to the patient with subsequent localized exposure to a light source of a specific wavelength, resulting in a cytotoxic response to oxidative damage. The present study intended to evaluate in vitro the type of induced death and the genotoxic and mutagenic effects of PDT alone and associated with cisplatin.

**Methods:**

We used the cell lines SiHa (ATCC® HTB35™), C-33 A (ATCC® HTB31™) and HaCaT cells, all available at Dr. Christiane Soares’ Lab. Photosensitizers were Photogem (PGPDT) and methylene blue (MBPDT), alone or combined with cisplatin. Cell death was accessed through Hoechst and Propidium iodide staining and caspase-3 activity. Genotoxicity and mutagenicity were accessed via flow cytometry with anti-gama-H2AX and micronuclei assay, respectively. Data were analyzed by one-way ANOVA with Tukey’s posthoc test.

**Results:**

Both MBPDT and PGPDT induced caspase-independent death, but MBPDT induced the morphology of typical necrosis, while PGPDT induced morphological alterations most similar to apoptosis. Cisplatin predominantly induced apoptosis, and the combined therapy induced variable rates of apoptosis- or necrosis-like phenotypes according to the cell line, but the percentage of dead cells was always higher than with monotherapies. MBPDT, either as monotherapy or in combination with cisplatin, was the unique therapy to induce significant damage to DNA (double strand breaks) in the three cell lines evaluated. However, there was no mutagenic potential observed for the damage induced by MBPDT, since the few cells that survived the treatment have lost their clonogenic capacity.

**Conclusions:**

Our results elicit the potential of combined therapy in diminishing the toxicity of antineoplastic drugs. Ultimately, photodynamic therapy mediated by either methylene blue or Photogem as monotherapy or in combination with cisplatin has low mutagenic potential, which supports its safe use in clinical practice for the treatment of cervical cancer.

## Background

Photodynamic Therapy (PDT) is a treatment modality considered an alternative to current treatments for cancer and infections. Briefly, it is based on the administration of a non-toxic dye (photosensitizing agent) to the patient with subsequent local exposure to a light source of specific wavelength [1], leading to the death of target cell via oxidative damage. PDT presents several advantages for the treatment of both tumors and infections, among which are noteworthy the minimum systemic adverse effects due to its double selectivity, resulting in localized treatment [2, 3].

Photodynamic therapy has been identified as a promising adjuvant therapy of conventional approaches due to its multiple mechanisms of action that individual drugs usually do not present. Therefore, the combination of therapies with different mechanisms of action may offer an advantage over monotherapies, since most diseases involve multiple and distinct pathologic processes [4]. Combining different approaches can result in benefits such as reaching several cellular targets, providing greater efficiency in destroying target cells and reducing doses of individual therapy components, with an overall improvement on therapeutic response and reduction of toxic effects [5].

With either a monotherapy or a combined modality, cancer treatment success is determined by the interaction of host immune cells and dying cancer cells, which can be achieved by merging the cytotoxic effect with immune stimulation capable of eliminating residual cells or possible micrometastasis [6, 7]. In fact, tumors responsive to PDT treatment are those presenting a great infiltrate of immune cells after the treatment [8]. Therefore, the ability of a cancer treatment to elicit host immune system stimulation via immunogenic cell death presents fundamental clinical relevance, since the anticancer immune response reinforces the therapeutic effect [7].

Besides inducing an immunogenic cell death, cancer therapies must not elicit additional mutations on surviving cells, since new mutations can result in cancer resistance to chemotherapy and, consequently, contribute to disease progression. Additionally, precaution is necessary regarding genotoxic damage, which could lead to the emergence of a secondary tumor, as an adverse effect of the anticancer agent on normal cells [9, 10].

PDT has the potential to attend all the requisites described above, either alone or combined with different approaches, as evidenced by a previous study of our group [5]. Then, we demonstrated that combining PDT with cisplatin significantly improved cell death, but information regarding the type of cell death induced by the combination treatment was lacking. Therefore, the aim of this study was to investigate the type of cell death induced by PDT and PDT combined with cisplatin, as well as their potential to induce mutations in surviving cells, using cervical cancer cell lines as a model.

## Methods

### Cell cultures

The cell lines used in this study were provided by Dr. Christiane Pienna Soares and were: SiHa (cervical carcinoma infected with HPV16; ATCC® HTB35™), C-33 A (cervical carcinoma not infected with HPV; ATCC® HTB31™), and HaCaT (spontaneously immortalized human keratinocytes; Addexbio Catalog #:T0020001). Cell lines were routinely checked for mycoplasma contamination. All cell lines were grown in a 1:1 mixture of Dulbecco’s Modified Eagle’s Medium (DMEM, Sigma Co., St. Louis, USA) and Ham’s Nutrient Mixture F10 (Sigma Co., St. Louis, USA) supplemented with 10% fetal bovine serum (FBS; Cultlab, Campinas, Brazil), 1X antibiotic/antimycotic solution (100 U/mL penicillin, 100 μg/mL streptomycin, 0.25 μg/mL amphotericin B; Sigma Co., St. Louis, USA) and 0.1 mg/mL kanamycin (Sigma Co., St. Louis, USA), which is referred to as “complete medium” hereafter. Cells were kept in 5% CO_2_ atm, 95% relative humidity and a constant temperature of 37 °C.

### Photosensitizers and drugs

All drugs and photosensitizers were prepared and stored protected from light.

Photogem (PG; Photogem LLC. Co., Moscow, Russia) and Methylene blue (MB; Sigma Co., St. Louis, USA) were dissolved in PBS and stored at −20 °C. Cisplatin (CisPT) was obtained as a 0.5 mg/mL solution (Tecnoplatin, Zodiac Produtos Farmaceuticos S/A, Brazil), stored at room temperature. Curcumin (CUR; Sigma Co., St. Louis, USA) was solubilized in dimethyl sulfoxide (DMSO) and stored at −20 °C. All drugs above were diluted to working concentrations prior to use. Doxorubicin (DOX) was obtained as doxorubicin hydrochloride powder (Cloridrato de doxorrubicina, Eurofarma, Brazil) and solutions were prepared in sterile distilled water immediately prior to use.

### Light source

Both light sources (630 and 660 nm) consisted of a compact LED array-based illumination system with a homogeneous illumination area and a cooling device, composed of 48 LEDs with variable intensities (IrradLED® – biopdi, Sao Carlos, SP, Brazil). The distance between the LED and the plate allowed an even distribution of light on each well. The power density of the incident radiation was measured using a power meter (Coherent®, Santa Clara, CA, USA).

### Photodynamic therapy and cisplatin treatment

Treatment groups are summarized in Table 1.

**Table 1:** Treatment groups for photodynamic therapy and cisplatin monotherapies and combination therapies

GROUPS	[PS]	[CisPT]	LIGHT DOSE
methylene blue (MB)	19.5 μM	0	0
Photogem (PG)	0.5 μM	0	0
MBPDT	19.5 μM	0	5.11 J/cm2
PGPDT	0.5 μM	0	2.76 J/cm2
cisplatin (CisPT)	0	41.6 μM	0
MBPDT + CisPT	19.5 μM	1.3 μM	5.11 J/cm2
CisPT + PGPDT	0.5 μM	1.3 μM	2.76 J/cm2

*PS*: photosensitizer; *MBPDT*: methylene blue-mediated photodynamic therapy; *PGPDT*: Photogem-mediated photodynamic therapy.

In the cisplatin-only group, cells were treated with 41.6 μM of cisplatin for 6 h. For the PG-photodynamic therapy (PGPDT) group, the cells were exposed to 630 nm at 2.76 J/cm^2^ after 2 h of incubation with 0.5 μM of PG. In the MB-photodynamic therapy (MBPDT) group, the cells were exposed to 660 nm at 5.11 J/cm^2^ after 20 min of incubation with 19.5 μM of MB. In the combination group, 1.3 μM of cisPT was administered for 6 h immediately after MBPDT or 6 h before PGPDT. All treatment conditions were determined based on previous studies of our group [5].

### Death profile assays

#### Fluorescence microscopy with Hoechst 33342 and propidium iodide

This assay was performed in a semi-automatic way at the IN Cell Analyzer 2000 (GE Healthcare Life Sciences, Pittsburgh, PA, EUA). For the assay, cells were cultivated in 96 wells plates in a cell density of 5,000 cells per well (5.0 × 10^4^ cell/mL) and, after 24 h of incubation at 37 °C and 5% CO_2_, treated according to the section *Photodynamic Therapy and cisplatin treatment*. After incubation time was complete, the plate was centrifuged at 2,500 rpm for 5 min at 4 °C. Media containing treatment was removed carefully and 100 μL of ice-cold PBS 1X were added to each well. The plate was centrifuged once more and PBS was removed. In a dark room, 100 μL of fluorochromes mix (HO 1 mg/mL – 15%; PI 1 mg/mL 25%, FDA 1 mg/mL 35%) prepared in ice-cold PBS was added to each well and plate was incubated at room temperature in the dark for 10 min. IN Cell Analyzer 2000 was set to capture 12 fields per well in each of the four wavelengths (bright field, green, blue and red filters) using a 20X objective. Images obtained were merged using IN Cell Analyzer 1000 Workstation 3.7 software (GE HealthCare, Pittsburgh, PA, EUA) and cells were counted manually. Five hundred cells were counted in each well and cell death was analyzed through the determination of live, apoptotic or necrotic cells based on cell morphology and fluorescence. The assay was performed in triplicates and was repeated three times.

#### Caspase-3 activity

Cells at a density of 2 × 10^5^ cell/mL were plated in 96 wells plates with a black bottom and incubated for 24 h at 37 °C and 5% CO_2_. Cells were treated according to the section *Photodynamic Therapy and cisplatin treatment* and placed over ice immediately after treatment period was over. Media containing treatment solutions were removed and each well received 100 μL of lysis buffer (50 mM Tris pH 7.4; 150 mM NaCl; 0.5% Triton X-100; EDTA 2 mM; DTT 5 mM). The plate was incubated on ice for 20 min and then 100 μL of substrate (20 μM Acetyl-Asp-Met-Gln-Asp-amino-4-methylcoumarin [Ac-DMQD-AMC]) prepared in lysis buffer were added to each well, in the dark. After substrate addition, the plate was read in a fluorometer (FLx800™ Fluorescence Reader, BioTek - Winooski, VT, USA; excitation 360/40 nm and emission 460/40 nm) by top reading after 30 s of gentle agitation. Reading was performed at 37 °C. Results were expressed as released 7-amino-4-methylcoumarin (AMC) concentration, based on the standard curve, which was prepared with decreasing concentrations of AMC beginning with 4 μM and ending in 0.0156 μM (2-fold dilutions). The assay was performed in triplicates and was repeated three times.

### Genotoxicity assays

#### Flow cytometry using anti-γH2AX antibody

Cells at a density of 2 × 10^5^ cells/well were plated in 24 wells plates, incubated for 24 h at 37 °C and 5% CO_2_, and treated according to section *Photodynamic Therapy and cisplatin treatment*. After treatment, cells were collected from the wells via trypsinization and centrifuged at 2.500 rpm for 10 min. Cells pellets were suspended in 1 mL of 4% paraformaldehyde and incubated for 10 min at room temperature. Cells were centrifuged again at 2.500 rpm for 10 min and washed once with 1X PBS. Each cell pellet was then suspended in methanol 70% (obtained with 30% 1X PBS) and stored at −20 °C until flow cytometry analysis. In the following step, cells were centrifuged at 2.200 rpm for 5 min and washed with 1X PBS. For permeabilization, cells were suspended in 0.025% Triton-X-100 and incubated for 5 min at room temperature, being once more centrifuged at 2.200 rpm for 5 min. Cells were incubated with anti-γH2AX antibody (2.5:1000) for 1 h at room temperature, without agitation. Again, cells were centrifuged at 2.200 rpm for 5 min and washed with 1X PBS. Then, cells were incubated with secondary antibody conjugated with Alexa Fluor® 488 (2.5:1000) for 30 min at room temperature, without agitation. Cells suspensions were centrifuged at 2.200 rpm for 5 min and washed with 1X PBS, being suspended in 60 μL of 1X PBS and analyzed by flow cytometry in a FACSCanto (BD - Becton, Dickinson, and Company, New Jersey, USA). The assay was performed in triplicates and was repeated three times.

#### Micronuclei assay

Five hundred cells were distributed in each well of a 96 wells plate. After 24 h of incubation at 37 °C and 5% CO_2_, cells were treated according to section *Photodynamic Therapy and cisplatin treatment*; media containing treatment substances were removed and replaced by complete medium. Cells were left to recover for 24 h. The next day, each well received 100 μL of complete medium containing 6 μg/mL cytochalasin B (Sigma Co., St. Louis, USA) and the plate was incubated for additional 24 h. After incubation, cells were fixed with absolute ethanol for 30 min and stained with 1 mg/mL fluorescein isothiocyanate (FITC) for 30 min, and 10 μg/mL Hoechst 33342 for 15 min. All procedures were performed at room temperature in the dark. Reading was performed in the IN Cell Analyzer 2000, which was set to capture 12 fields per well in each of the three wavelengths (bright field, green and blue filters) using a 20X objective. Images obtained were fused using IN Cell Analyzer 1000 Workstation 3.7 software (GE HealthCare, Pittsburgh, PA, EUA) and cells were analyzed manually. The assay was performed in triplicates and was repeated three times.

#### Clonogenic survival

Cells were plated in duplicates at a density of 150 cells/well in six wells plates and incubated until attached to the bottom of the well (3 h at 37 °C and 5% CO_2_; adhesion was confirmed by microscopic observation). Once adhered, cells were treated according to section *Photodynamic Therapy and cisplatin treatment* and, after each treatment time, the medium was removed and replaced by complete medium. The plates were incubated at 37 °C and 5% CO_2_ for 7 days, without media exchange. After the 7 days, the medium was removed and cells were washed with 1X PBS, fixed with a mixture of methanol, acetic acid and water (1:1:8, respectively) for 30 min and stained with crystal violet for 15 min. Established colonies were analyzed using a magnifying lens (16X magnification). Colonies containing < 50 cells were not considered and results were expressed in plating efficiency (PE) and survival fraction (SF), according to Franken et al. [11]. The assay was performed in duplicates and was repeated three times.

### Statistical analysis

Data were expressed as the mean plus standard deviation (SD) and were analyzed by one-way ANOVA with Tukey’s posthoc or Kruskal-Wallis with Dunn’s posthoc test using GraphPad Prism® Version 5.01 software (GraphPad Software Inc., La Jolla, CA, USA). Differences were considered to be significant when *p* < 0.05. The acceptable coefficient of variation was less than or equal to 25%.

## Results

**Fig. 1:**
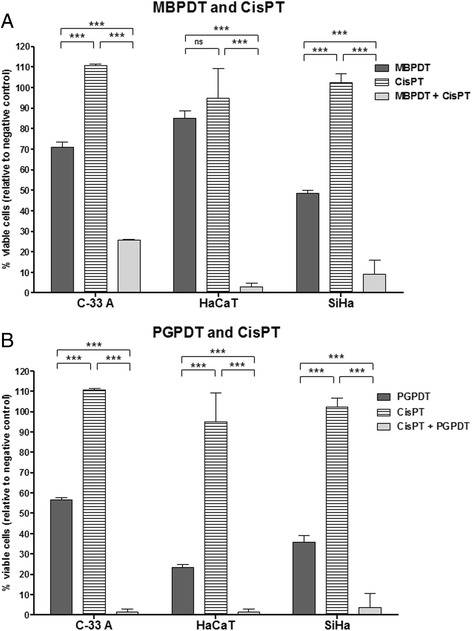
Comparison of cytotoxic effects of Photodynamic Therapy and cisplatin, as monotherapies and combined. **a** cell lines were treated with MBPDT (19.5 μM; 5.11 J/cm^2^), cisplatin (1.3 μM for 6 h) and combined therapy (MBPDT + CisPT, using the same parameters of monotherapies). **b** cell lines were treated with PGPDT (0.5 μM; 2.76 J/cm^2^), cisplatin (1.3 μM for 6 h) and combined therapy (CisPT + PGPDT, using the same parameters of monotherapies). It is observed a great reduction in cell viability caused by combined therapies, compared to monotherapies. Asterisks indicate the statistical differenc. Columns represent the average of four independents quadruplicates and bars represent the standard deviation. ANOVA one-way, with Tukey posthoc. *p < 0,05; **p < 0,01; ***p < 0,001

In previous studies of our group, we observed that both the photodynamic therapy mediated by methylene blue (MBPDT) and Photogem (PGPDT) were effective in reducing cell viability with cytotoxicity being dependent on the light dose, for all three cell lines analyzed (C-33 A, HaCaT and SiHa). Cisplatin was less effective over the three cell lines compared to PDT (Fig. 1). However, the combination cisplatin + PDT had a synergistic effect and caused greater cell death in all conditions tested (Fig. 1). The sequence of treatment application (PDT + cisplatin or cisplatin + PDT) influenced the response and effectiveness depended on the photosensitizer: for MBPDT we found that PDT prior to cisplatin was more effective; on the other hand, for PGPDT the efficiency increased when cisplatin treatment was performed before PDT [5]. Therefore, the aim of this study was to investigate the type of cell death induced by PDT and PDT combined with cisplatin, as well as their potential to induce mutations in surviving cells, using cervical cancer cell lines as a model.

### Cell death profile characterization

#### Fluorochrome exclusion assay: Hoechst 33342 and propidium iodide

**Fig. 2:**
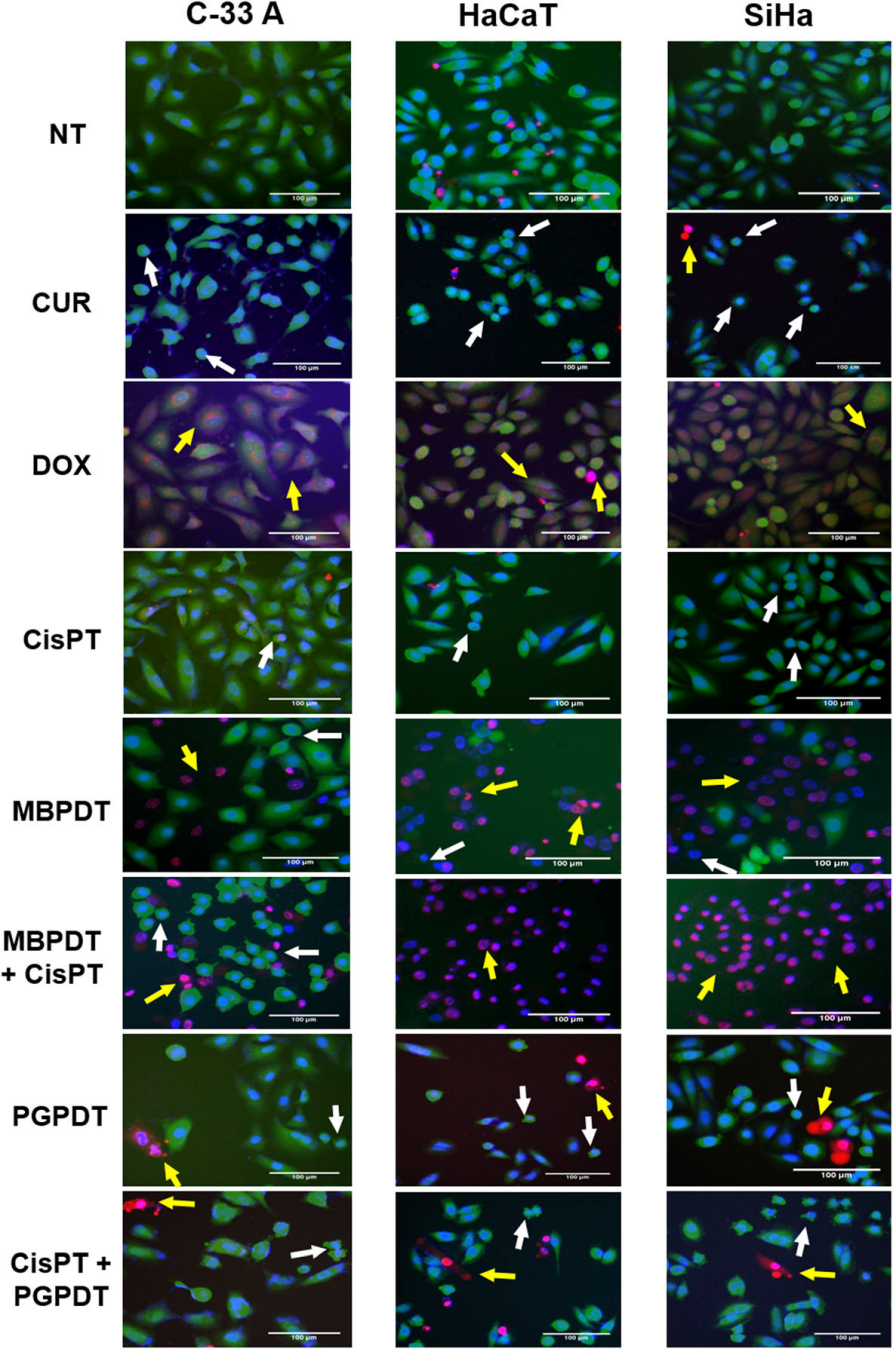
Fluorescence images obtained from IN Cell Analyzer, using Hoechst 33342, propidium iodide and fluorescein diacetate. Cell lines C-33 A, HaCaT and SiHa were submmited to Hoechst 33342, propidium iodide and fluorescein diacetate staining after treatments (CUR [25 μM for 6 h]; DOX [50 μg/mL for 6 h]; CisPT [41.6 μM for 6 h]; MBPDT [19.5 μM MB + 5.11 J/cm^2^ LED 660 nm]; MBPDT + CisPT [19.5 μM MB + 5.11 J/cm^2^ LED 660 nm + 1.3 μM CisPT for 6 h]; PGPDT [0.5 μM PG + 2.76 J/cm^2^ LED 630 nm]; CisPT + PGPDT [1.3 μM CisPT for 6 h + 0.5 μM PG + 2.76 J/cm^2^ LED 630 nm]). White arrows indicate representative apoptotic cells and yellow arrows indicate representative necrotic cells. 20X objective. NT: non-treated; CUR: curcumin; DOX: doxorubicin; CisPT: cisplatin; MBPDT: photodynamic therapy mediated by methylene blue; PGPDT: photodynamic therapy mediated by Photogem

**Fig. 3:**
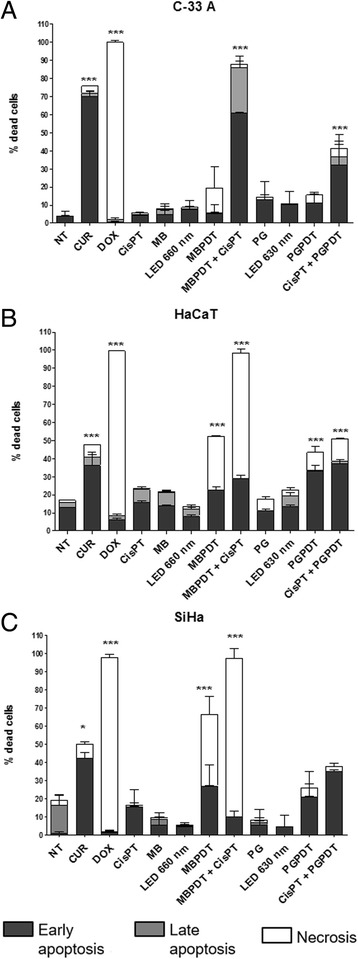
Cell death profile induced by each treatment, according to cell line. Cell lines C-33 A (panel **a**), HaCaT (panel **b**) and SiHa (panel **c**) were submmited to Hoechst 33342, propidium iodide and fluorescein diacetate staining after treatments (CUR [25 μM for 6 h]; DOX [50 μg/mL for 6 h]; CisPT [41.6 μM for 6 h]; MB [19.5 μM for 20 min]; LED 660 nm [5.11 J/cm^2^]; PG [0.5 μM for 2 h]; LED 630 nm [2.76 J/cm^2^]; MBPDT [19.5 μM MB + 5.11 J/cm^2^ LED 660 nm]; MBPDT + CisPT [19.5 μM MB + 5.11 J/cm^2^ LED 660 nm + 1.3 μM CisPT for 6 h]; PGPDT [0.5 μM PG + 2.76 J/cm^2^ LED 630 nm]; CisPT + PGPDT [1.3 μM CisPT for 6 h + 0.5 μM PG + 2.76 J/cm^2^ LED 630 nm]). Columns represent the average of three independent assays and bars represent standard deviation. Asterisks indicate the statistical difference, relative to negative control. Kruskal-Wallis, with Dunn’s post-hoc. *p < 0,05; **p < 0,01; ***p < 0,001. NT: non-treated; CUR: curcumin; DOX: doxorubicin; CisPT: cisplatin; MB: methylene blue; MBPDT: photodynamic therapy mediated by MB; PG: Photogem; PGPDT: photodynamic therapy mediated by PG

As expected, cells treated with doxorubicin and curcumin induced, primarily, necrosis and apoptosis, respectively, with the percentage of necrotic or apoptotic cells varying according to the cell line (Figs. 2 and 3), with C-33 A being the most sensitive one (Figs. 2 and 3a). When cells were treated only with LED light sources or photosensitizers in the dark we did not observe significant cell death in comparison with the negative control cells (Figs. 2 and 3a-c).

MBPDT induced a greater amount of cell death in SiHa cells, compared to C-33 A and HaCaT, accordingly to our previous results. However, opposing previous studies [12–14], MBPDT caused cell death with predominant necrotic morphology, with about 2/3 of dead cells identified as necrotic and 1/3 presenting morphology of typical apoptosis (Fig. 2).

The combination therapy of MBPDT and cisplatin kept the death profile, with a predominance of necrotic cells for SiHa and HaCaT, but cell death percentage was even higher than that with both MBPDT and cisplatin as monotherapies (Figs. 2, 3b-c). C-33 A also presented higher levels of cell death when submitted to the combined therapy in comparison to monotherapies; however, there was a predomination of typical apoptosis morphology, a death profile more similar to that of cisplatin monotherapy (Figs. 2 and 3a).

When cell lines were treated with PGPDT we also observed cells with both necrotic and apoptotic morphologies, but with a predominance of the apoptotic type (Figs. 2 and 3a-c). Contrary to what was observed for MBPDT + cisplatin, combined therapy of cisplatin and PGPDT did not induce a greater percentage of cell death when compared to PGPDT monotherapy, except for C-33 A, which again presented a distinct behavior from the other two cell lines. When compared to cisplatin as monotherapy, cisplatin + PGPDT was capable of inducing increased rates of cell death for all cell lines evaluated.

#### Caspase-3 activity assay

The Nomenclature Committee on Cell Death (NCCD) recommends that apoptosis, or other types of cell death, has to be demonstrated by more than one methodology as a procedure to eliminate artifacts. Therefore, to complement and confirm the results of cytomorphological fluorochromes exclusion assay described above, we conducted a caspase-3 activity assay.

With the only exception of positive control (curcumin), none of the treatments induced detectable capase-3 activation. Concerning negative control (NC), MB, PG, LED630 and LED660 the result confirmed the observed non-induction of cell death; similarly, doxorubicin did not induce caspase activation as expected, since it promotes death by necrosis, which is independent of caspases. Although cisplatin had promoted apoptosis as monotherapy, the slight toxic effect observed over the cell lines used in this study generated a few apoptotic cells that probably did not produce a detectable signal.

Treatments with photodynamic therapy, either as monotherapy or combined with cisplatin, did not activate caspase-3. Such result indicates the cell death caused by PDT in the conditions employed in this study is caspase-independent, which agrees with previous studies that associated caspase-independent cell death (CICD) with increased amounts of reactive oxygen species [15–17], which are responsible for PDT’s mechanism of action.

### Genotoxicity

#### Flow cytometry with anti-γH2AX

Table 2 shows the results of γH2AX labeling obtained for C-33 A, HaCaT e SiHa cells. Average fluorescence intensity is directly proportional to the frequency of DNA’s double strand breaks induced by the treatments [18–20]. Cells treated with light, the photosensitizers or cisplatin only did not suffer significant DNA damage, with the concentrations employed, comparing to non-treated cells.

**Table 2:** Detection of H2AX phosphorylation via flow cytometry

	Average fluorescence intensity		
Treatments	C-33 A	SiHa	HaCaT
No treatment	1256	1405	1545
Secondary antibody	1197	1465	1391
CisPT	1503	1575	1526
LED660	1318	1533	1591
MB	1211	1401	2069
MBPDT	2471**	2531**	2842*
MBPDT + CisPT	3362**	3115**	10547***
LED630	1376	1489	1669
PG	1452	1414	1677
PGPDT	1386	1428	1695
CisPT + PGPDT	1444	1534	10547

Cell lines C-33 A, HaCaT and SiHa were submmited to flow cytometry using anti-γH2AX monoclonal antibody after treatments (CisPT [41.6 μM for 6 h]; MB [19.5 μM for 20 min]; LED 660 nm [5.11 J/cm^2^]; PG [0.5 μM for 2 h]; LED 630 nm [2.76 J/cm^2^]; MBPDT [19.5 μM MB + 5.11 J/cm^2^ LED 660 nm]; MBPDT + CisPT [19.5 μM MB + 5.11 J/cm^2^ LED 660 nm + 1.3 μM CisPT for 6 h]; PGPDT [0.5 μM PG + 2.76 J/cm^2^ LED 630 nm]; CisPT + PGPDT [1.3 μM CisPT for 6 h + 0.5 μM PG + 2.76 J/cm^2^ LED 630 nm]). Asterisks indicate the statistical difference relative to non-treated samples. Kruskal-Wallis, with Dunn’s post-hoc (ns: non significative; *p < 0,05; **p < 0,01; ***p < 0,001). *CisPT*: cisplatin; *MB*: methylene blue; *MBPDT*: photodynamic therapy mediated by MB; *PG*: Photogem; *PGPDT*: photodynamic therapy mediated by PG

On the other hand, it is possible to observe that MBPDT induced pronounced DNA double strand breaks in all cell lines, in an opposite way of PGPDT, which did not cause DNA damage. Similarly, the combined therapy MBPDT + cisplatin induced a higher number of breaks than the combined therapy PGPDT + cisplatin, which resulted in a damage index comparable to that of PGPDT.

To verify the mutagenic potential of the therapies evaluated here, treatments that caused DNA damage were analyzed regarding their ability to induce mutations, which was done using the micronuclei assay, described below.

#### Micronuclei assay

**Fig. 4:**
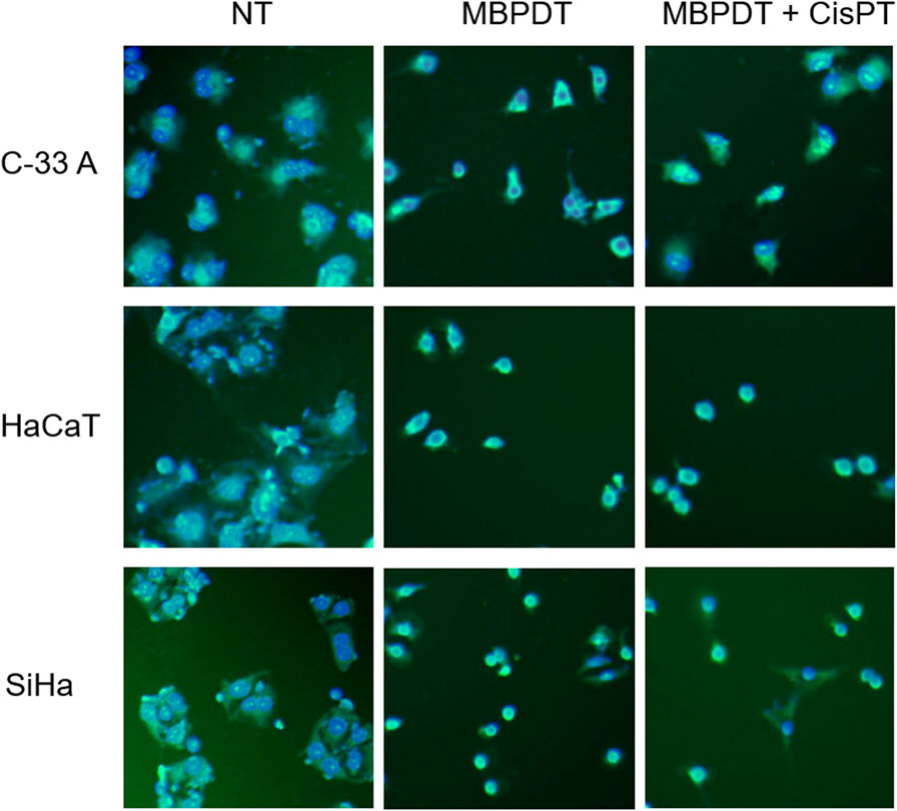
Micronuclei assay. Representative images of micronuclei assay. Yellow arrows indicate a few binucleated cells. Images captured using IN Cell Analyzer 2000, 20X objective. NT: non-treated; MBPDT: photodynamic therapy mediated by methylene blue (19.5 μM MB + 5.11 J/cm^2^ LED 660 nm) MBPDT + CisPT: MBPDT followed by cisplatin treatment for 6 h (1.3 μM CisPT)

For this assay, we considered only the treatments that induced DNA double strand breaks detected in the flow cytometry with anti-γH2AX, i.e., MBPDT and MBPDT combined with cisplatin. However, we could not perform micronuclei counting after those treatments given that this damage is detected only when cells execute mitosis. Thus, it is mandatory that treated cells have kept their cellular division capacity after treatment. Nevertheless, as can be seen in Fig. 4, we could not find a single binucleated cell after MBPDT or MBPDT + cisplatin treatments, unlike the negative control cells.

**Fig. 5:**
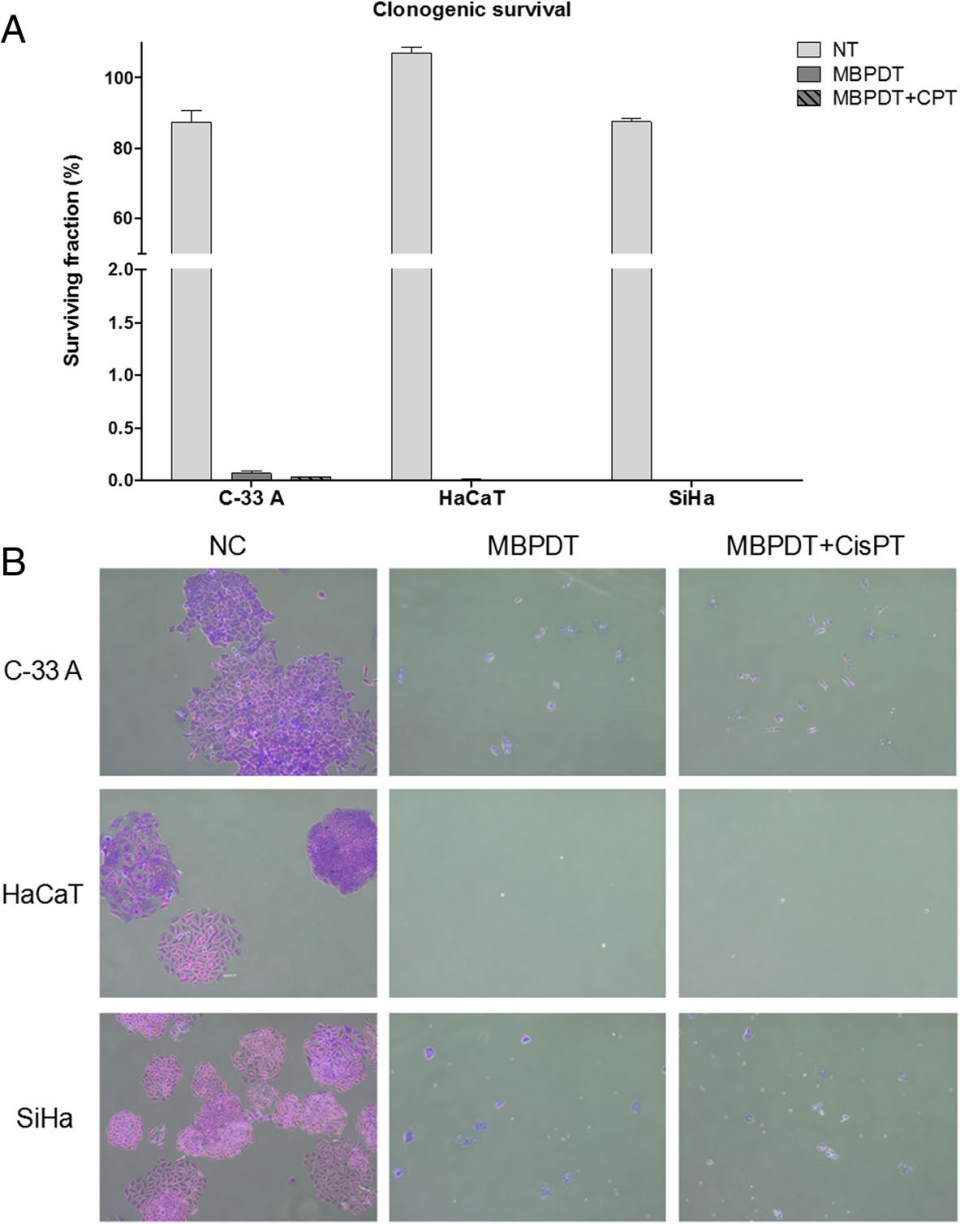
Clonogenic survival. **a** Survival fraction of cell lines treated or not with MBPDT or MBPDT + CisPT. Columns represent the average number of colonies in each condition (a colony was considered so when presenting more than 50 cells), after three independent assays. Bars indicate the standard deviation. Asterisks indicate the statistical difference, relative to negative control. Kruskal-Wallis, with Dunn’s posthoc (*p < 0,05; **p < 0,01; ***p < 0,001). **b** Representative images of colonies formed in each treatment condition. Cristal violet staining; 10X objective (Olympus CKX31 microscope). NT: non-treated; MBPDT: photodynamic therapy mediated by methylene blue (19.5 μM MB + 5.11 J/cm^2^ LED 660 nm) MBPDT + CisPT: MBPDT followed by cisplatin treatment for 6 h (1.3 μM CisPT)

In order to confirm that such outcome was indeed a result of the loss of cell division capacity, we performed a clonogenic survival assay. It was confirmed that cells have lost their normal cell division capability since the few surviving cells failed to divide even after 7 days of incubation (Fig. 5).

## Discussion

In a previous study of our group [5], we employed the MTT assay to assess cell viability of C-33 A, HaCaT, and SiHa after treating those cell lines with PDT mediated either by methylene blue or Photogem, alone or combined with cisplatin. The cell viability of the combined therapy groups was significantly lower compared to monotherapies. The sequence of treatments (PDT + cisplatin or cisplatin + PDT) was important and had different results when varying the PS, but combination therapy resulted in an enhanced anticancer effect regardless of the treatment protocol, enabling the use of cisplatin at a concentration 12.5-fold lower compared with cisplatin-only treatment. In the following step, we sought to investigate how the cells were dying and whether or not the therapies could induce mutations.

Regarding the type of induced cell death, MBPDT induced the typical morphology of necrosis (Figs. 2 and 3). Contrary of what is described in previous studies [12–14], MBPDT caused cell death with predominantly necrotic morphology, with about 2/3 of dead cells identified as necrotic and 1/3 presenting morphology typically apoptotic (Fig. 2c). Such difference can be due to the incubation time with the photosensitizer prior to LED irradiation. Differently of our work, most of the previous cell culture studies employed protocols with dark incubation of 1 h [13, 21]. The incubation for 20 min employed in this work may have been insufficient for MB to reach the cellular targets needed to trigger cell death by apoptosis. Therefore, if MB was restricted to the cytoplasmic membrane proximities at the moment of irradiation, it is possible that reactive oxygen species (ROS) and singlet oxygen formed might have induced peroxidation of membrane lipids [22], which could have led to loss of membrane integrity and favored cell death by necrosis [23].

Cisplatin predominantly induced apoptosis, and combined therapy induced different rates of apoptosis- or necrosis-like depending on the cell line, but always with a higher percentage of dead cells than monotherapies (Figs. 2 and 3). Those results highlight the synergistic effect between PDT and cisplatin and corroborate the data obtained in the cytotoxicity assays (Fig. 1). Moreover, the different cell death profiles observed among the three cell lines after treatment with combined therapies indicate that the cytotoxic effects elicited by each individual therapy are dependent on the cell type.

Morphology alterations induced by PGPDT were most similar to apoptosis (Figs. 2 and 3). Several studies have shown that Photogem can induce both types of cell death [24, 25]. Therefore, our results were as expected and were in concordance with the literature. In this study, in general, cell death rates obtained for PGPDT, both as monotherapy and combined with cisplatin, were unexpectedly low, taking into account the results obtained in the cytotoxicity assay (Fig. 1). It is possible that PGPDT induces cellular damages that culminate in cell death in later times, which could have generated the low estimative of the post-treatment death rate in this assay due to the incubation times employed.

Members of the cysteine protease family, caspases have a central role in the coordination of stereotyped events occurring during apoptosis [26]. Caspases are activated in response to various cell death stimuli and lead to disassembly of the cell by proteolysis of hundreds cellular targets [27, 28]. According to the phase of the apoptotic process in which they operate, caspases are subdivided into initiator (caspases −8, −9 and −10), which connects the intrinsic or extrinsic cell death stimuli to the following caspases in the pathway, so-called effectors (caspase - 3, −6 and −7), which activate other cellular factors [26]. Caspase-3, for example, promotes cleavage of PARP1 (poly (ADP-ribose) polymerase 1) and internucleosomal DNA fragmentation, one of the hallmarks of apoptosis [29]. Thus, detection of caspase-3 activity is a hallmark of classical apoptosis occurrence by the caspase-dependent pathway.

In this study, both MBPDT and PGPDT did not activate capase-3 in any of the cell lines, indicating that those treatments induced caspase-independent cell death (CICD).

CICD is defined by some authors as the cell death that occur when stimuli that would usually cause apoptosis fails to activate the caspase [29]. Although typical events of caspase action are not present, like phosphatidylserine externalization and nuclear fragmentation, other characteristics of CICD resembles those of apoptosis, such as permeabilization of the mitochondrial outer membrane, loss of proliferation capacity and nuclear condensation [29, 30]. However, cells undergoing CICD may present a wide variety of characteristics, depending mainly on the initial stimuli and cell type [29]. On Fig. 2 we can see treatment protocols involving MBPDT resulting in cell death with morphology similar to necrosis, while the protocols involving PGPDT produced cell morphologies more similar to those of apoptotic cells, which demonstrates that initial stimuli seem to be more important than cell type to determinate the characteristics of the death process that the cells will undergo.

Specialized literature brings a huge discussion concerning the type of cell death induced by photodynamic therapy and the subsequent antitumor immune response triggered. It is commonly accepted that cell death via necrosis prompts acute inflammatory response and, therefore, has an immunogenic role. Likewise, it is known that apoptotic cells are cleared from the tissue in a silent way, without leading to an inflammatory environment or an immunological response [31, 32]. However, the idea of immunogenic apoptosis was raised by several authors and has gained strength in recent years [1, 33, 34].

The concept of caspase-independent cell death is recent and, therefore, there are little enlightening studies on the subject. It is not known how the tissue responds to those cells, how they are removed from the tissue and what is the role of CICD in the development of antitumor immunity [15, 29]. However, since it presents characteristics both of apoptosis and necrosis, it is possible that CICD plays an important role in the development of antitumor immunity. In fact, it was observed that some cell lines that die in a caspase-independent manner are highly immunogenic [35].

Besides conflicting ideas, there is a consensus over the fact that the generation of an intense acute inflammatory response after photodynamic therapy exerts a positive role in immune system activation and, by doing so, triggers the establishment of a lasting antitumor immunity, with potential to control possible recurrences of the primary malignant tumor and micrometastasis [1, 7].

Therefore, treatment protocols that favor tumor cell death by both apoptosis and necrosis, as the protocols shown in this work, have a great potential to induce antitumor immunological response: necrotic cells would provoke the necessary inflammatory response to attract defense cells, and apoptotic cells being phagocytized by professional antigen presenting cells would trigger the development of specific antitumor immunological response. Nevertheless, more studies are required to determine if the type of cell death observed for the PDT treatments of this work would be the key for the establishment of a lasting and effective antitumor immunity.

MBPDT, either as monotherapy or in combination with cisplatin was the unique therapy to induce significant damage to DNA (double strand breaks) in the three cell lines (Table 2). Several studies have evaluated the genotoxic potential of photodynamic therapy, using a variety of photosensitizers, light sources and cell lines. Induction of DNA damage by PDT was identified in all works available so far, with singlet oxygen being the species directly related to alterations observed in the DNA. The type and extension of DNA damage vary accordingly to the PS and its concentration, light dose and cell line [36–39].

Results obtained in this work corroborate the previous studies mentioned, particularly concerning MBPDT. The great extension of DNA damage induced by this therapy was accompanied by increased cell death, mainly with a necrotic profile, either as monotherapy or combined with cisplatin. Preceding studies have shown that extensive DNA damage caused by oxidative stress lead to cell death by necrosis: after genomic injury PARP-1 is activated and catalyzes the hydrolysis of NAD+, depleting it from cellular context and causing an energetic failure. That process results in caspase-independent cell death with necrotic features [40–45].

Besides double and single strand breaks, PDT can induce DNA cross-linking, sister chromatid exchange and base oxidation in the DNA, particularly in guanine residues; various studies suggest the formation of the 8-hydroxydeoxyguanosine residue after PDT mediated by methylene blue [36–38, 46]. Thus, we can speculate that, although PGPDT did not cause DNA double strand breaks, other types of DNA damage could be generated by this treatment. This is an interesting hypothesis to be tested in further studies.

To determine if the observed genotoxic action could be mutagenic, we performed the micronuclei (MN) formation assay. MN can originate from acentric chromosomal fragments (missing the centromere) or whole chromosomes incapable of migrating to cell poles during anaphase of cell division. Therefore, micronuclei represent a permanent damage that has been transmitted to the cell’s offspring [47]. There was no mutagenic potential for the damage induced by MBPDT, given that the few cells that survived the treatment have lost their clonogenic capacity (Figs. 4 and 5).

Therefore, although MBPDT and MBPDT + cisplatin treatments induce extensive DNA damage, the few cells that survive the treatment cannot propagate the acquired mutations because they do not have any proliferative capacity. By knowing the mechanism of cell death caused by the combined therapy one would have more certainty that this new approach will not cause additional mutations to the cancer cells or their surrounding normal cells, which could complicate the treatment. In addition, although it requires further studies, combining PDT with cisplatin may have a positive effect on the development of specific antitumor immunity, preventing the recurrence of the primary tumor.

Since cisplatin have been used to treat patients with cervical cancer for decades [5], and PDT have been used to treat early cervical cancer in clinical studies ([48–52]; among others), we believe it is completely possible to reproduce our results in vivo. By doing so, the cisplatin dose administered to the patients would be reduced due to its association with PDT because cells are being attacked by two different mechanisms, diminishing the required dose to trigger cell death. This has a very important impact on reducing adverse effects provoked by antineoplastic drugs since less deleterious effects are observed when lower doses are administered.

## Conclusions

Our data showed that, with the methods employed, MBPDT and PGPDT induced cell death to the three cervical cancer cell lines analyzed, with predominant morphological characteristics of necrosis and apoptosis, respectively. However, none of the treatments activated caspase-3.

We also observed that MBPDT, both as monotherapy and as combined therapy with cisplatin, was able to induce DNA double-strand breaks in the three cell lines evaluated, while PGPDT did not. Despite its genotoxicity, MBPDT was not mutagenic since it inhibited the proliferation capacity of surviving cells.

When combining PDT and cisplatin the advantages of each individual treatment are enhanced. Low doses of cisplatin administered prior to PGPDT optimized the result, and MBPDT sensitized tumor cells for cisplatin action. Taken together, those results elicit the potential of such combined therapy in diminishing the toxicity of antineoplastic drugs.

Ultimately, photodynamic therapy mediated by either methylene blue or Photogem as monotherapy or in combination with cisplatin has low mutagenic potential and possibly high immunogenic potential, characteristics that support its safe use in clinical practice for the treatment of cervical cancer.

## Abbreviations

AMC: 7-amino-4-methylcoumarin
CICD: Caspase-independent cell death
cisPT: Cisplatin
CUR: Curcumin
DOX: Doxorubicin
DTT: Dithiothreitol
EDTA: Ethylenediaminetetraacetic acid
FDA: Fluorescein diacetate
HO: Hoechst 33342
LED: Light emitting diode
MB: Methylene blue
MBPDT: PDT mediated by MB
MN: Micronuclei
MTT: 3-(4,5-Dimethylthiazol-2-yl)-2,5-Diphenyltetrazolium Bromide
PBS: Phosphate-buffered saline
PDT: Photodynamic therapy
PG: Photogem
PGPDT: PDT mediated by PG
PI: Propidium iodide
PS: Photosensitizer
